# Enhanced visible light photodegradation activity of RhB/MB from aqueous solution using nanosized novel Fe-Cd co-modified ZnO

**DOI:** 10.1038/s41598-018-29025-1

**Published:** 2018-07-16

**Authors:** Neena D., Kiran Kumar Kondamareddy, Han Bin, Dingze Lu, Pravin Kumar, R. K. Dwivedi, Vasiliy O. Pelenovich, Xing-Zhong Zhao, Wei Gao, Dejun Fu

**Affiliations:** 1 Key Laboratory of Artificial Micro- and Nano-Materials of Ministry of Education, Wuhan University, Wuhan, China; 2 Hubei Key Laboratory of Nuclear Solid Physics, School of Physics and Technology, Wuhan University, Wuhan, China; 3 Department of Chemical & Materials Engineering, University of Auckland, Auckland, New Zealand; 4 Department of physics, Veltech Rangarajan Dr. Sagunthala R&D Institute of science and technology, Avadi, Chennai, Tamilnadu India; 5 Inter Department of Physics, Xi’an Polytechnic University, Xi’an, China; 6 Inter University Accelerator Centre (IUAC), Aruna Asaf Ali Marg, New Delhi, India; 7 Department of Physics, Christ Church College, Kanpur, India

## Abstract

A series of novel Fe-Cd co-doped ZnO nanoparticle based photocatalysts are successfully synthesized by sol-gel route and characterized using scanning electron microscopy (SEM), energy dispersive X-ray emission (EDX), transmission electron microscopy (TEM), X-ray diffraction (XRD), UV-Vis spectroscopy, X-ray photoelectron spectroscopy (XPS), and Brunauer-Emmett-Teller (BET) techniques. The photocatalytic activity of ZnO nanoparticles doped with various atomic weight fraction of Fe and Cd has been investigated under visible light irradiation using the Methylene Blue and Rhodamine B dye in aqueous solution. The FeCd (2%):ZnO (ZFC-1) exhibit the highest photocatalytic activity in terms of rate constant as K_MB_ = 0.01153 min^−1^ and K_RhB_ = 0.00916 min^−1^). Further, the re-usability of the ZFC-1 photocatalyst is studied which confirms that it can be reused up to five times with nearly negligible loss of the photocatalytic efficiency. Moreover, the role of photoactive species investigated using a radical scavenger technique. The present investigations show that the doping concentration plays significant role in photocatalytic performance. The visible light absorption shown by Fe-Cd co-doped ZnO nanoparticles is much higher than that of undoped body probably due to co-doping, and the charge carrier recombination is decreased effectively which yields a higher photocatalytic performance. The mechanism for the enhancement of photocatalytic activity under visible light irradiation is also proposed.

## Introduction

The industrial effluents contain, organic pollutants such as dyes, phenoxyaniline, phenols and their derivatives which are acutely toxic and difficult to biodegrade^1^. These contaminants are genotoxic and may disrupt endocrine systems even at low concentrations, and hence the human health is at high risk. The de-colourisation and complete mineralization is difficult because of the complex structure of the dyes and high recalcitrance to its degradation. Major studies have been carried out to develop new fascinating materials for dye removal. Rhodamine B (RhB: C_28_H_31_ClN_2_O_3_) and Methylene blue (MB: C_16_H_18_N_3_SCl) dyes are highly detrimental to the ecosystem and thus, pose menace to the animals and human beings^2^. To minimize the risk of pollution from these toxic chemicals and to enable the recycling of water resource, it is necessary to properly treat the industrial waste water prior to its drainage. Various techniques, which have been extensively exploited for dye removals are chemical oxidation, coagulation, adsorption and photocatalysis, etc^3,4^. Photocatalysis is an inexpensive vital way for controlling the current environmental pollution at the large- scale. In particular, the photocatalysts with semiconducting nanostructures have attracted much attention owing to their large aspect ratio, providing larger active portion (or area) for the photocatalytic reaction^5,6^. Furthermore, they are superior because of their excellent chemical stability, easy preparation, and low cost^7^. Hence, researchers have recently developed a lot of semiconductor metal oxide nano-photocatalysts, including Bi_2_O_3_, TiO_2_, ZnO, and Fe_2_O_3_, which are useful for keeping a comfortable climate for the human beings^8-11^. Among these semiconductor metal oxide photocatalysis, TiO_2_ and ZnO are found quite suitable in photocatalytic process owing to their nontoxicity, wide bandgap, and high photosensitivity^12–15^.TiO_2_ has been studied and used extensively. However, ZnO seems to be a better candidate because of its unique physical and chemical properties, low cost and environmental stability. Furthermore, the efficiency of ZnO is greater than that of TiO_2_ in photocatalytic degradation for a few dyes in aqueous solution. Nevertheless, to meet the practical applications, the rapid recombination of photo-generated electrons and holes has to be effectively suppressed to enhance the quantum yield and the photocatalytic efficiency of ZnO and the absorption of light in the visible light range should be intensified as well^16–18^. To date, several technologies have been developed to suppress the recombination of photo-induced charge carriers, a common process of photocatalytic reactions by the integration of the photocatalysts with electron scavenging species including organic molecules, metal and metal oxides. The doping of ZnO with transition metals has significant influence on its photocatalytic efficiency. The cadmium doped nanostructure photocatalyst degrades methylene blue up to 80% in 180 mins under visible light irradiation^19^. R. Saravanan *et al*. reported that CdO/ZnO nanowires degrade methylene blue in 6 h under visible light irradiation^20^. Zhang *et al*. revealed that the use of Fe/ZnO nanowires is much better than that of P 25 against methyl orange^21^. It has been found that 2% Fe doped ZnO degraded methyl orange up to 80.79% in 210 mins of sun-light irradiation. The Fe doped ZnO degrades methylene blue in 4 h in sun light^22^. It is believed that a synergism occurs between the two dopants in capturing electrons and thus co-doping can greatly enhance photocatalytic activity by capturing more electrons. Furthermore, for dye removal and having higher photocatalytic activity, the performance of Fe and Cd co-doped ZnO is far better than that of bare/intrinsic one The co-doing is found more effective to photocatalytic activity with low and moderate atomic weight fractions of Fe and Cd, respectively.

To date, there has been no report on the synthesis of Fe-Cd co-doped ZnO photocatalyst with efficient electron-hole separation ability. Herein, we report a successful attempt for the synthesis of the Fe-Cd co-doped ZnO nanoparticles via sol- gel method, and the photocatalytic activity of these nanoparticle photocatalysts were explored by measuring their ability of degradation of RhB and MB dyes. The influence of different parameters such as amount of photocatalyst, different concentrations of dopant and dye on the efficiency was also investigated. The Fe-Cd co-doping leads to increase the active sites on the surface photocatalysts and results in to the enhanced photocatalytic activity. Finally, we succeeded in re-using Fe-Cd co-doped ZnO catalyst for 5 times without any loss of activity.

## Results and Discussion

### Morphology, chemical state and microstructure of the (Fe-Cd) doped ZnO photocatalysts

**Figure 1:**
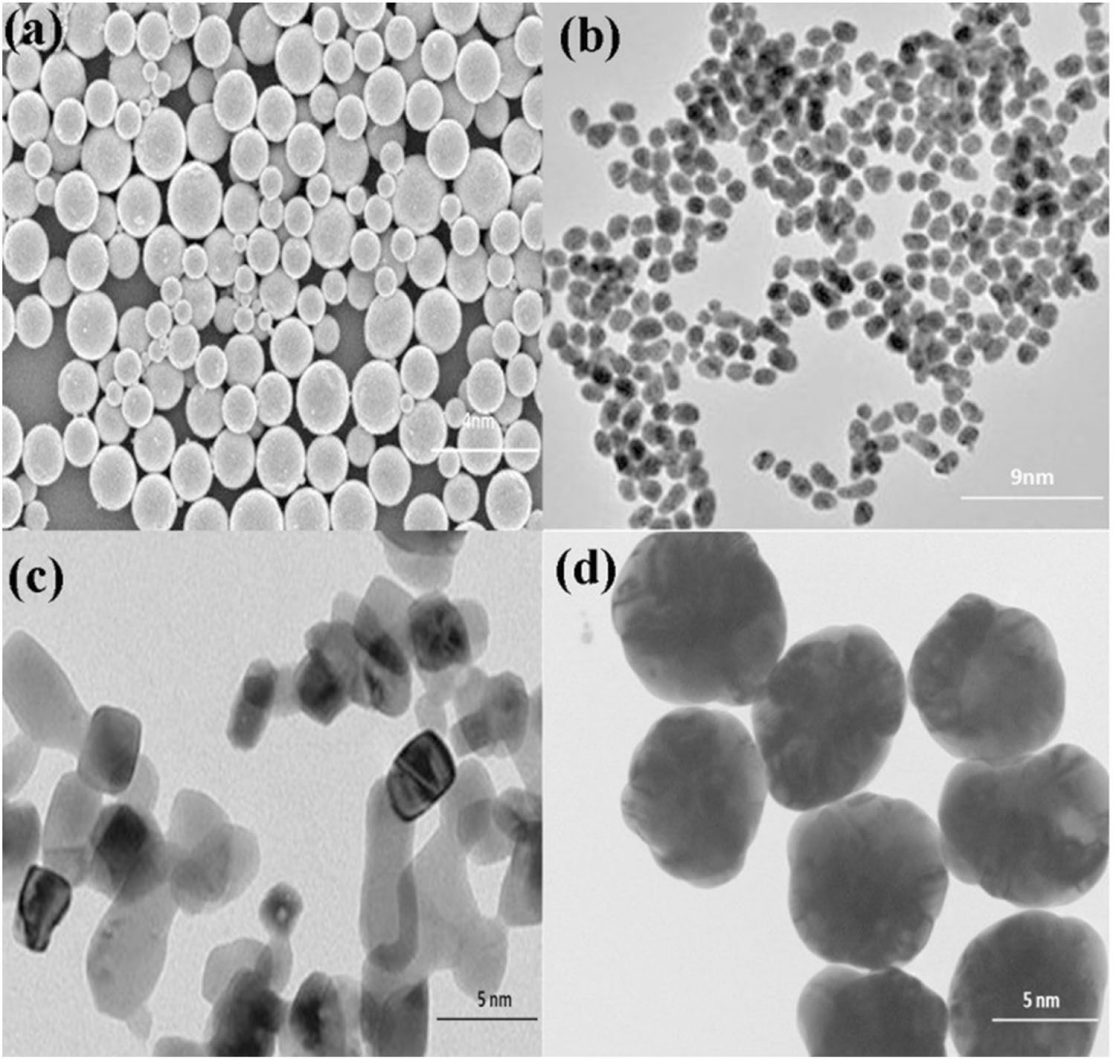
TEM micrographs for nanoparticles of (**a**) ZnO, (**b**) ZFC-1, (**c**) ZFC-2 and (**d**) ZFC-3 photocatalysts.

**Figure 2:**
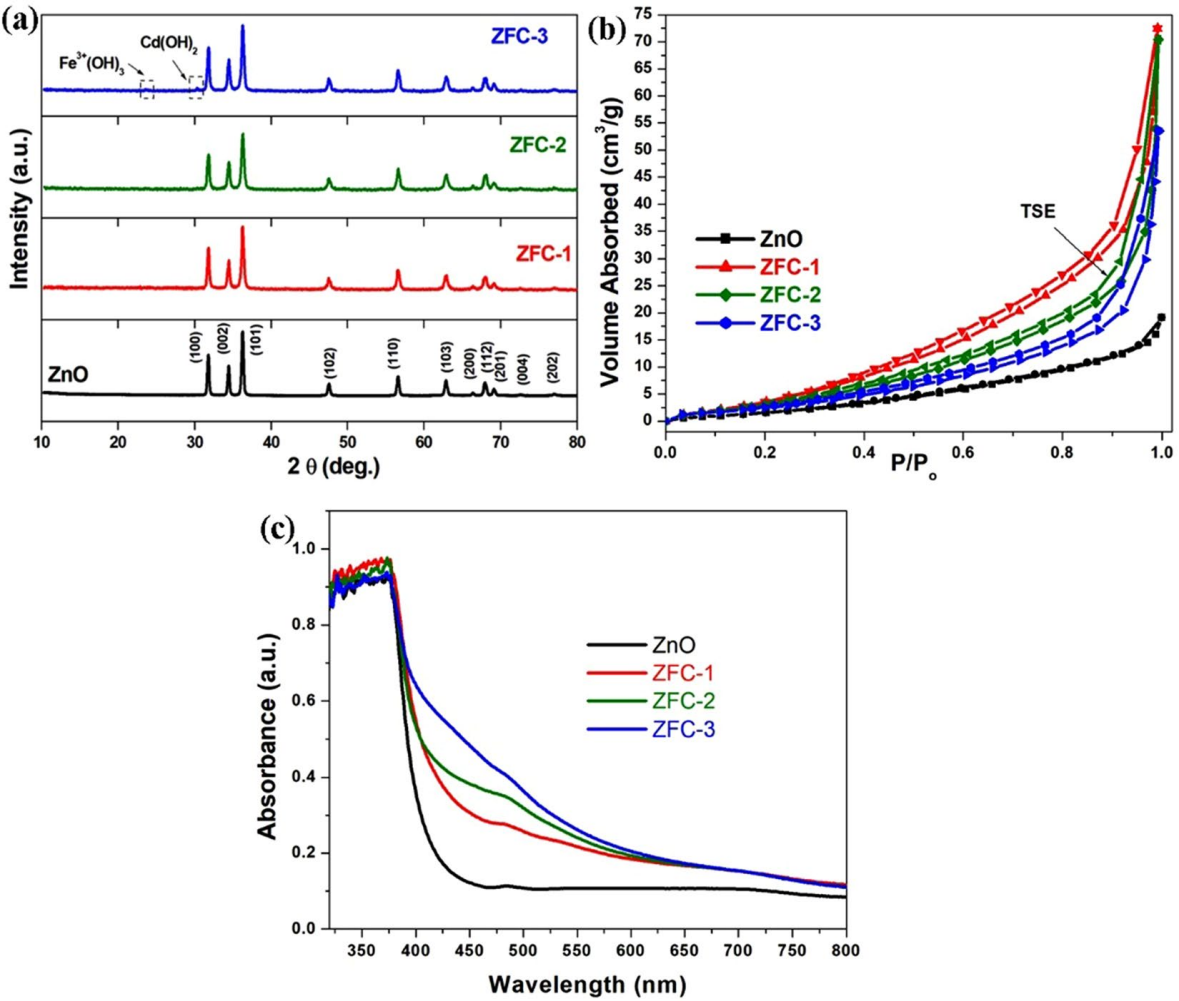
(**a**) X- ray diffraction patterns, (**b**) Nitrogen adsorption–desorption isotherms, and (**c**) UV-Vis absorption spectra of pure and doped ZnO samples.

The morphology of pure and Fe-Cd co-doped ZnO samples is studied by TEM and scanning electron microscopy (See Figure S1, Supporting Information) and is displayed in Fig. 1. The spherical nanoparticles are clearly visible which show almost uniform size distribution, smooth surface and grain boundaries with small agglomeration. A closer observation of SEM images of all samples reveals that the particles size of pure ZnO is more than that of ZFC-x samples, which is in good agreement with XRD results (see Fig. 2). Micro structural analysis of TEM images, indicates that the most nanoparticles are spherical in shape with smooth surfaces. TEM images show that synthesized nanoparticles have clear and well defined grain boundaries which can affect the physical characteristics of samples.

Figure 2(a) shows the XRD patterns of the samples ZnO, ZFC-1, ZFC-2 and ZFC-3. All XRD peaks were indexed using the standard JCPDS file for ZnO (JCPDS #36–1451). The observed peaks correspond to the hexagonal wurtzite structure of crystalline ZnO. The characteristic peaks of α-Fe, α-Fe_2_O_3_, γ-Fe_2_O_3_ or Fe_3_O_4_ were not detected in any of the samples. A systematic increase in the full width at half maximum (FWHM) and a decrease in the intensity of the diffraction peaks of ZnO were observed with increasing concentration x of Fe/Cd. These changes demonstrate that the increasing Fe-Cd content results into a reduction in the average crystallite size (D*v*) and a loss of crystallinity of the wurtzite ZnO phase. The average crystallite sizes of ZnO estimated from XRD data are 42.8 nm, 34.8 nm, 30.3 nm and 31.6 nm for samples ZnO, ZFC-1, ZFC-2 and ZFC-3, respectively. These results indicate that Fe-Cd incorporation in the ZnO structure inhibits crystallite growth, perhaps by modifying the rate of nucleation during the ZnO crystallization. Since the ionic radii of Fe^3+^, Cd^2+^and Zn^2+^ are different (Fe^3+^  = 0.49 Å, Cd^2+^  = 0.74 Å, Zn^2+^  = 0.60 Å)^23,24^ the addition of Fe-Cd dopants to ZnO results in to a significant shift in (101) peak of ZnO. The samples are found to be of single phase and no trace of other impurities was observed within the sensitivity of the technique up to 4% Fe-Cd. However, for sample ZFC-3, some secondary phases are observed at 23.54° and 30.33°, which ascribed to Fe^3+^(OH)_3_ (JCPDS # 46–1436) and Cd(OH)_2_ (JCPDS # 20–0179), respectively.

The isotherms of gaseous N_2_ adsorption–desorption [Fig. 2(b)] were recorded to evaluate the porosity of pure and co-doped ZnO. The typical type IV curve accompanied with H3 type hysteresis loop was observed in the isotherms, indicating the predominance of mesopores^25^. All of the samples show sloping of the adsorption and desorption boundary curves. Due to the tensile strength effect (TSE), the residual condensate can leave the pores, resulting the formation of abrupt region for the desorption curve of the doped ZnO. The ZFC-1 exhibits strongest TSE. Moreover, the observation of hysteresis in isotherms denotes the presence of porous 3D intersection network^26^. It can be seen that the doping of ZnO transition metal ions results an increase in the area of the hysteresis loop which can be explained on the basis of development of the porous network due to the formation of small nanocrystals.

The specific BET surface area of ZnO, ZFC-1, ZFC-2, and ZFC-3 is found to be 5.487, 12.348, 10.229 and 8.677 m^2^/g, respectively. In general microporosity leads kinetic problems such as restricting desorption brunch to meet the absorption brunch. The present ZFC-x nanoparticles show high adsorption rate at relative pressures P/P_0_ close to 1.0, suggesting the formation of large macrospores and mesopores^26^. The XRD and XPS studies reveal that there are very less metal (Fe and Cd) and other impurities formed on the surface of mesoporous ZnO and hence the metal and secondary oxide phases cannot block the pores. The changes observed for pore volume and surface area may be attributed to the formation of small average crystallites because of incorporation of metal ions in the ZnO lattice. The specific surface area corresponds to effective charge transport is optimum for ZFC-1 which can be responsible for its enhanced photocatalytic performance.

The separation of photo-induced charge carriers is closely related to the band gap which is measured from the UV-Vis absorption curves shown in Fig. 2(c). The spectra for Fe-Cd based ZnO have a small shoulder (hump) in the range of 460 to 490 nm. The d-d crystal- field transitions between multiplets of 3d^5^ configuration of the high spin Fe^3+^ substituting for Zn^2+^ under the influence of a tetrahedral crystal field results such a weak hump^27^. Therefore, this hump is attributed to the presence of Fe^3+^ at an octahedral sites^28^. The intensity of hump changes as some of Fe^3+^ ions that present at tetrahedral sites shifts to octahedral sites with increasing (Fe-Cd) content. The peak around ~500 nm and a weak shoulder at ~420 nm might be due to the existence of very weak impurity phases of MFe_2_O_4_ (M = Zn^2+^ or Cd^2+^), which were not detected by XRD^29^. Furthermore, the position of absorption edge for Fe-Cd co-doped ZnO nanoparticles slightly shifts towards the longer wavelengths relative to that of pure ZnO nanoparticles. The observed red-shift up on co-doping of ZnO nanoparticles indicates the incorporation of Fe/Cd into ZnO matrix. The band gap values of the samples were measured using Tauc relation and the corresponding the plots are given in supplementary material (See Figure S2). The band gaps for the samples are found to reduce gradually with the concentration of the dopants. The reduced bandgap of ZnO (Figure S2) and enhanced absorbance which is quite suitable for photocatalytic activity^30^. A possible cause of reduced band gap in doped material can be s-d and p-d exchange interaction, which results in a positive and negative shift of the valance and conduction band edges, respectively^31^.

**Figure 3:**
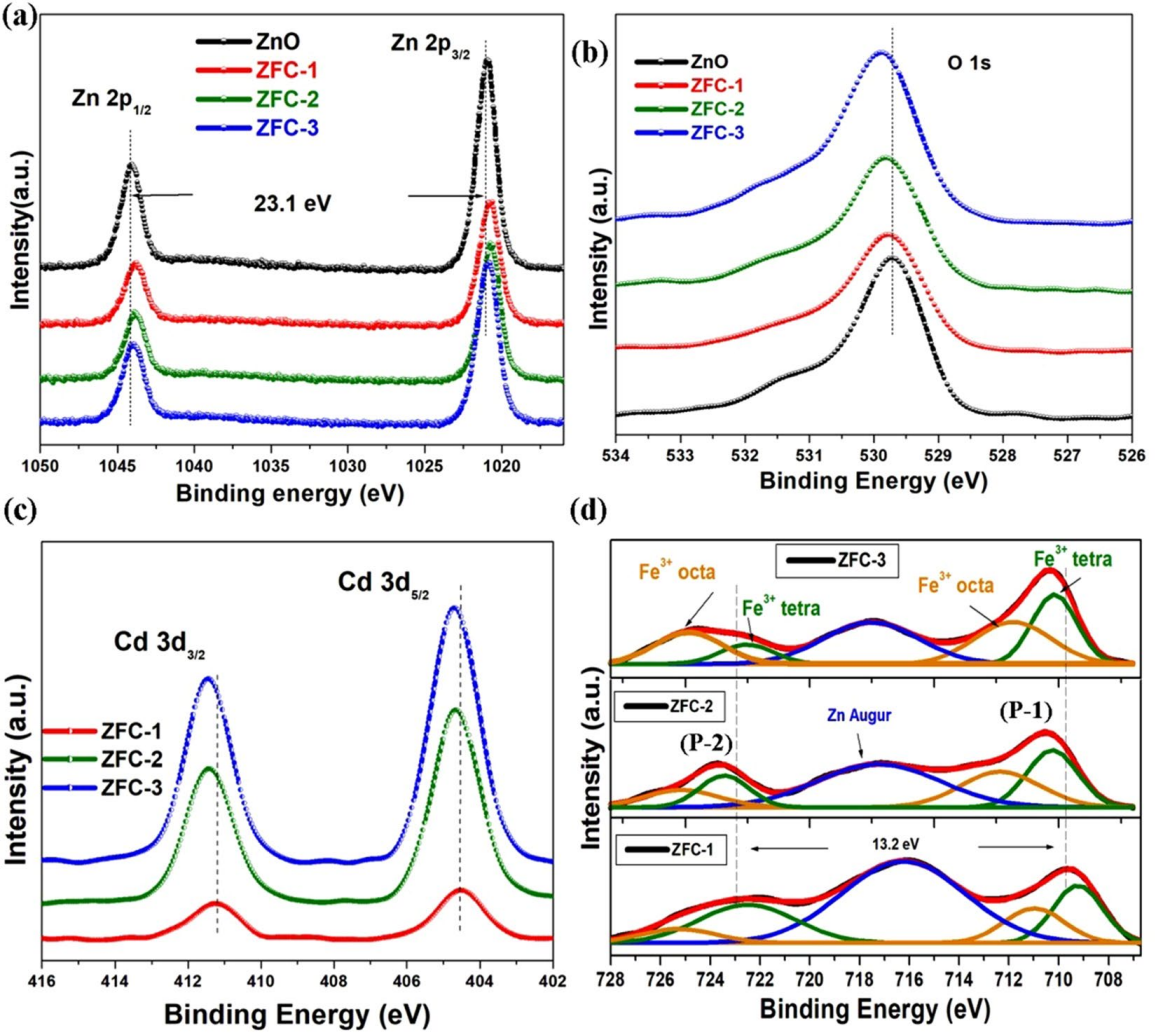
(**a**) HR Zn 2p core-electron region, (**b**) O 1s core-electron region, (**c**) Cd 3d core-electron region, and (**d**) Fe 2p core-electron region of all samples.

The chemical states and elemental composition of the present nanostructures were evaluated by X- ray photoelectron spectroscopy (XPS). The XPS spectra of Zn 2p core levels for ZFC-x samples are presented in Fig. 3(a). The peak correspond to Zn 2p_3/2_ for undoped and ZFC-x samples is located at around 1020.7-1020.9 eV, with 23.1 eV spin-orbit splitting (ΔS). From the observed BE values, we can realize that the Zn ions preserve the bivalent state in tetrahedral sites for all samples. No other oxidation states were seen. A reduction in relative area of the peaks Zn 2p_3/2_ and 2p_1/2_ with the concentrations of Fe-Cd co-dopants is clearly seen which implies that most of the Fe and Cd ions are incorporated substitutionally at host Zn sites. The slight chemical shift towards lower binding energy side is observed upon Fe and Cd substitution. This shifting might be due to the enhanced valance electron density of Zn^2+^ ions, which indicates the interaction of Fe and Cd ions/atoms with Zn atoms of host materials^32^. The O 1s core level spectra are shown in Fig. 3(b). These profiles exhibit broad asymmetric features, which can be deconvoluted into two components, suggesting the presence of two different chemical environments for oxygen ions (Figure S3).

The relatively stronger component (O-1) at lower binding energy side in the range of (529.67-529.84) eV is ascribed to O_2_^−^ ions surrounded by Zn^2+^ atoms of the wurtzite structure at tetrahedral sites. The second peak (530.73-530.86 eV, O-2) is attributed to the formation of surface chemisorbed −OH groups, suggesting the existence of loosely bound oxygen atoms near the surface region of ZnO nanoparticles^33,34^. It is found that the intensity of the O-1 peak decreases, with increasing the concentration of Fe and Cd up to 2%, which indicates an enhanced bulk oxygen-deficient regions. The increased FWHM of O-2 peak with (Fe-Cd) content suggests the formation of higher oxygen vacancies and other defects on the surface of ZnO nanoparticles. The formation of surface –OH groups can also be contributed by the reduction of crystallite size up on co-doping. It is also observed that the co-doping of ZnO causes a chemical shift in the binding energies of O1s peaks which suggests an effective interaction between the dopants Fe and Cd and ZnO lattice. Further the shift may be attributed to the presence of a very small amount of an impurity phase. Figure 3(c) represents two strong peaks located at around 404 eV and 411 eV, which can be assigned to the binding energy of Cd 3d_5/2_ and Cd 3d_3/2_, respectively. The binding energy of Cd 3d_5/2_ peak (404.5 eV) for ZFC-2 sample is similar to Cd^2+^ for ZnCdO alloy films, confirming the presence of Cd-O^35^. It was found that the Cd occupy Zn sites in ZnO to form present photocatalysts, which is consistent with XRD studies. The intensity of the Cd 3d peak increases gradually and shifts to higher binding energy (from 404.5 eV to 404.6 eV) with the concentration of dopant. This might be ascribed to the structural deformation and enlargement up on the partial substitution of Zn^2+^ by Cd^2+^ ions for the photocatalysts^36^.

Figure 3(d) reveals high-resolution XPS spectra of Fe 2p core level for ZFC-x samples. The broad and asymmetric profile has been deconvoluted into two components, which indicate the presence of two kinds of Fe^3+^ with nonequivalent chemical environments. The corresponding peaks are designated as (P-1) and (P-2), respectively with splitting ΔS in the range of 13.2-13.5 eV. These observations exclude the possibility of formation of metallic Fe clusters with 3^+^ oxidation state from the doped Fe ions, which is also evidenced by XRD results. The first component, at lower BE (P-1) is attributed to Fe^3+^ ions of tetrahedral site, while the second component (P-2) resulted from the Fe^3+^ located at octahedral sites^37,38^. The area of the second component (P-2) increases with the (Fe−Cd) content, suggesting more Fe^3+^ ions occupy octahedral sites. The remarkable component at ~717.5 eV in all samples is ascribed to Auger peak of Zn. In short, the surface hydroxyl groups of the photocatalysts play a vital role in its adsorption and photocatalytic studies, and abundant hydroxyl groups enhance the photoactivity. The shift in XPS spectra may be due to the formation of new bonds/impurities, which results in to the improved surface acidity and hence the polar organic dyes might be easily adsorbed on the surface of the sample^29,39^.

### Photocatalytic performance

#### Influence of doping concentration

**Figure 4:**
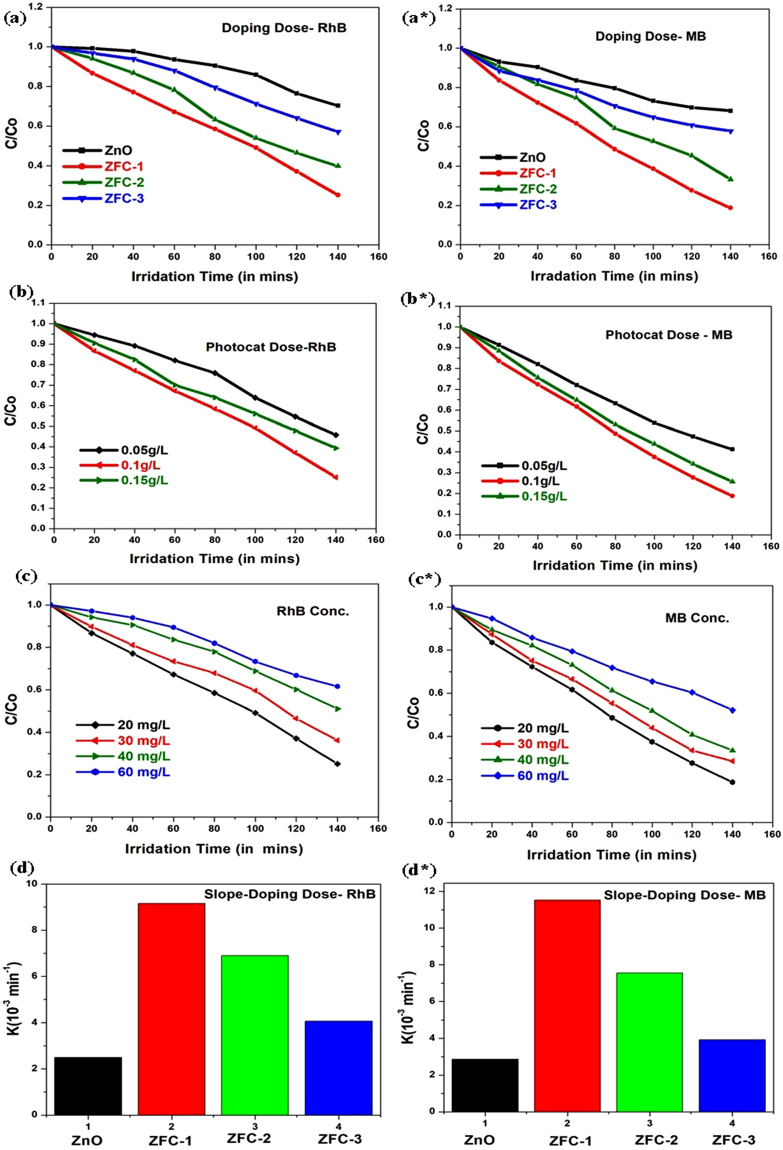
Influence on Photodegradation of RhB and MB aqueous solution with different (**a**-a*) doping concentration, (**b**-b*) photocatalysts mass, and (**c**-c*) dye concentration. (**d**-d*) degradation rate of pollutant.

The photocatalytic activities of pure and doped ZnO samples were performed using the photocatalytic degradation of MB and RhB under visible light irradiation (λ ≥ 420 nm) and the performance is depicted in Fig. 4(a-a*). The degradation rate of pure ZnO sample is very slow under visible-light illumination, which is mainly attributed to the photosensitization of dye. As per the literature, Fe/ZnO and Cd/ZnO nanostructures reveal their ability to remove organic pollutants^20,40^.However, when Fe species and Cd species are co-doped into ZnO site to form the ZFC-x, its photocatalytic activity enhances in comparison to Fe/ZnO, Cd/ZnO and ZnO samples^22,41,42^. Under visible light irradiation, all ZFC-x photocatalysts show photocatalytic performance higher than that of pure ZnO. The degradation rate constant (*k)* is increased step-by-step from pure ZnO, ZFC-3, and ZFC-2 to ZFC-1 nanoparticles photocatalyst (Fig. 4d-d*). The ZFC-1 photocatalyst reveals the highest photocatalytic activity, in degrading RhB and MB by 76% and 82% in 140 mins, respectively. The rate constant *k* values of ZFC-1 for MB and RhB are 0.0115 and 0.00916 min^−1^ respectively and it is ~3.7 times as higher than that of pure ZnO. Hence particularly we infer that the introduction of Fe and Cd can synergistically enhance the photocatalytic activity of ZnO nanoparticles. In addition, the apparent rate constant *k* of the ZFC-1 nanoparticles for RhB dye is 1.33 times and 2.25 times of that of ZFC-2 and ZFC-3, respectively (See Table 1). The rate constant *k* of the ZFC-1 nanoparticles for MB dye is 1.52 times and 2.93 times of that of ZFC-2 and ZFC-3, respectively. These results demonstrate that the introduction of (Fe-Cd) could facilitate charge transfer, enhancing the photocatalytic activity only up to the optimum doping (2% Fe-Cd) level. The adsorption under dark condition for ZFC-1 is also presented in Figure S4 (See Supporting Information) to show the attainment of balance between adsorption and desorption.

**Table 1:** Rate constants for both dyes with various parameters.

		Rate constant (min^−1^) for RhB	Rate constant (min^−1^) for MB
Photocatalysts	ZnO	0.00250	0.00286
	ZFC-1	0.00916	0.01153
	ZFC-2	0.00690	0.00756
	ZFC-3	0.00407	0.00393
Photocat. Loading	5 mg	0.00553	0.00778
	10 mg	0.00916	0.01153
	15 mg	0.00653	0.00927
Dye Conc	20 mg/L	0.00916	0.01158
	30 mg/L	0.00678	0.00913
	40 mg/L	0.00467	0.00782
	60 mg/L	0.00363	0.00459

### Influence of photocatalyst loading

The effect of the photocatalyst dosage on the degradation rate of RhB and MB using ZFC-1 photocatalyst was examined by altering the amount of photocatalyst from 0.05 g/L to 0.15 g/L (Fig. 4(b-b*)). The degradation efficiency of ZFC-1 photocatalyst was demonstrated for 140 mins, and constant initial concentration of 20 mg/L for RhB and MB, each. The rate constants *k* determined for the degradation of 20 mg/L RhB and MB solution (100 mL) are given in Table 1. However, the degradation efficiency tended to decrease as the photocatalyst dosage was further increased. As reported in literature^43–45^, there is an adoptable dosage of photocatalyst for achieving high efficiency .It is well known that the available surface area or the number of active sites on the surface of sample increases with increasing the amount of photocatalyst. Therefore, the number of superoxide and hydroxyl radicals also increases. Moreover, the degradation efficiency decreased due to the opacity of the suspension for photocatalyst dosage above optimum value. The interception of the light provides increased scattering of the light and photocatalyst surface becomes unavailable for light absorption^46^.

### Influence of dye concentration

The impact of initial concentration of RhB and MB (20, 30, 40 or 60 mg/L) on its degradation under visible light irradiation was also performed and shown in Fig. 4(c-c*). It is observed that the decomposition rate of the dye depends on its initial concentration and the photo-degradation rate suppresses with increase in dye concentration. It is believed that the rate of degradation is correlated with the formation of reactive oxygen species (ROS) such as hydroxyl radicals (·OH) and super-oxide (·O_2_^−^) radicals and the reaction rate of these ROS with the organic dyes such as RhB and MB. Furthermore, an increase in the amount of dye adsorbed on the catalyst surface by increase of the initial concentration of dye, causes a reduction of the catalyst activity. The results confirm that the more coverage of active sites by RhB and MB ions reduces the concentration of ROS. Another possible explanation for this trend is the influence of visible light irradiation on organic dyes. A significant amount of light is absorbed by the pollutant molecules (at a high dye concentration) rather than by the photocatalyst providing the reduction in the formation of ROS as well as the photocatalytic activity^47,48^.

**Figure 5:**
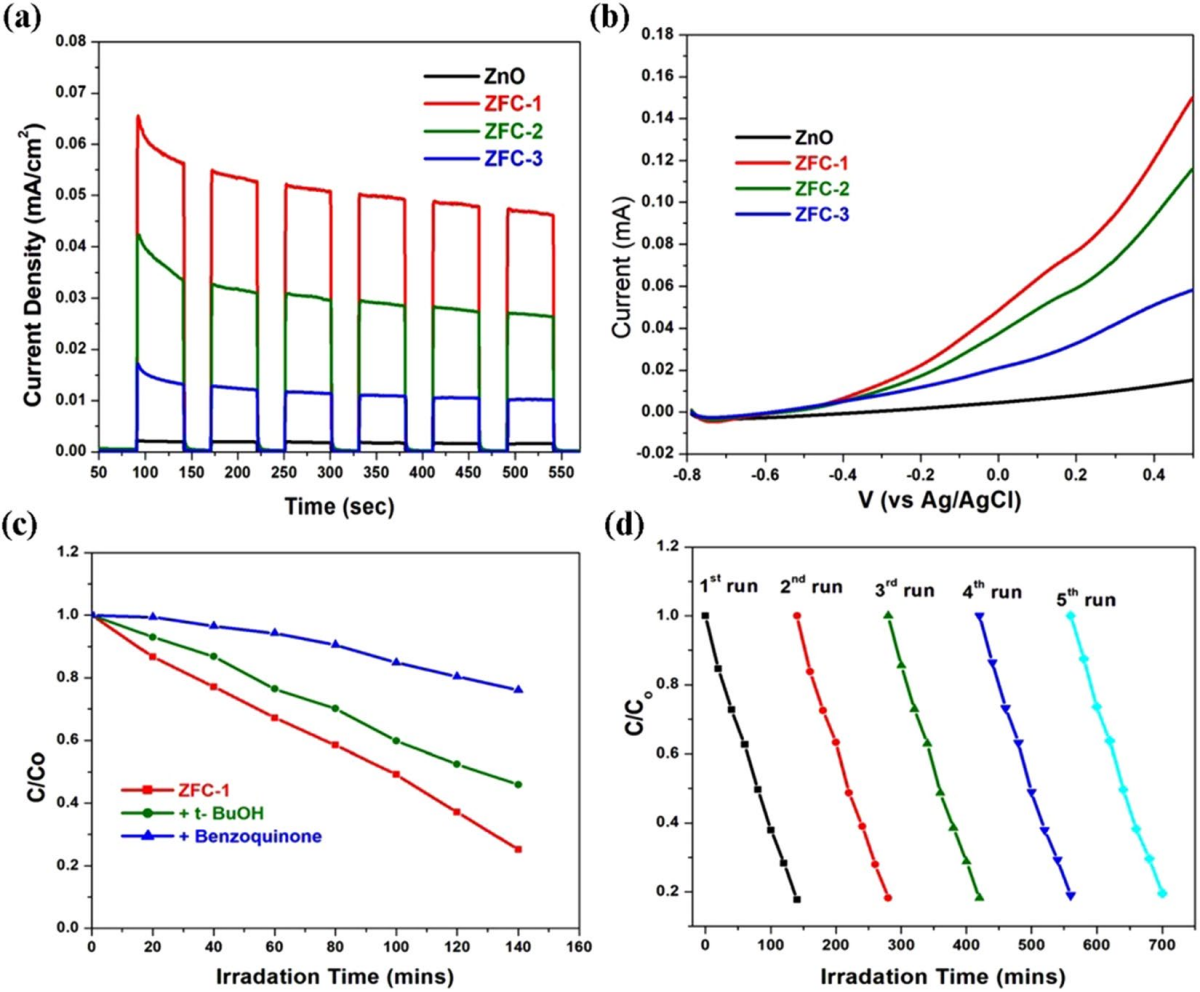
(**a**) Surface photocurrent, (**b**) Linear sweep volumetric (LSV) spectra, (**c**) Influence of t-BuOH and benzoquinone used as ·OH and ·O_2_^−^ scavengers, respectively, on the photocatalytic activity of ZFC-1, and (**d**) Reusability of ZFC-1 for the cyclic degradation with initial concentration of RhB dye.

The recycle test was performed for five cycles using the best photocatalyst ZFC-1 to confirm the reusability of the photocatalyst (Fig. 5d). From the results of repetitive photocatalysis tests, it was observed that sample ZFC-1 showed excellent photocatalytic efficiency for the degradation of RhB dye. Further, Fe-Cd co-doped ZnO nanostructures seem to be excellent reusable photocatalysts for the degradation of toxic organic dyes in water under visible light irradiation.

### Photogenerated Charge separation

The photo-electrochemical experiments were carried out to demonstrate the electronic behavior for ZnO, and ZFC-x which is displayed in Fig. 5(a). After the first clock for each light-on and light-off cycle in all the electrodes gave fast and uniform photocurrent response. This fast response indicates that the charge transport process in the electrodes is very quick. The pure ZnO nanoparticles provide almost no photocurrent response under the visible light irradiation. With the doping of (Fe-Cd), the photocurrent density (PCD) of ZFC-x samples increases initially and then, decreases. The PCD attains maximum value for 2% doping of Fe-Cd. It is found that the photocurrent density of ZFC-1 is much higher than that of other ZFC- x, suggesting that optimal co-doping can effectively improve the transport of charge carriers and also separation efficiency of photo-generated electron–hole pairs. The present results suggest that the excess amount of Fe-Cd co-dopants would develop recombination centers for the photo-generated electron-hole pairs and thus suppress the separation of photo-generated electron-hole pairs^49^. To further investigate the visible light response of the ZFC-x samples, we also carried out Linear Sweep Volumetric (LSV) measurements in dark and under visible light illumination at a scan rate of 10 mV/s and the results were presented in Fig. 5(b). There is almost no evidence of photocurrent for ZnO electrode, which indicates that there is no electrochemical oxidation of water in the surface of ZnO electrode under visible light illumination. The photocurrent of ZFC-x gradually enhances from that of pure ZnO, indicating the effective improvement on the photo-conversion efficiency with co-doping. The enhancement in photocurrent is typically related to improved rate of photo-induced carrier transport as well as photo-generated electron−hole pair separation^38,50^. On the basis of these results, we infer that the co-doping of ZnO by Fe-Cd can serve as an effective material for the enhancement of the photocurrent. The formation of superoxide (·O_2_^−^) and hydroxyl radicals (·OH) under photocatalytic conditions and their role in the degradation process of RhB or MB has been studied indirectly with the help of appropriate quenchers of these species (see Fig. 5c). In these studies, a comparison is made between the degradation curves of ZFC-1 and those recorded after addition of quenchers to the initial solution by keeping all conditions identical. The t-BuOH and benzoquinone were used as scavenger for ·OH radicals and quencher for ·O_2_^−^, respectively. From the results of photocatalysis process, it was noted that the rate of degradation decreased in presence of t-BuOH (300 μL in 50 mL of the RhB or MB solution), but the photocatalytic activity was not significantly suppressed. In the presence of benzoquinone (5 mg in 50 mL of the dye solution), the transformation of dye is completely suppressed suggesting that ·O_2_^−^ radicals anions play a vital role in the degradation process. During this experiment, the absorption peak slightly increases which may be attributed to the polymerization of benzoquinone into oligohydroquinones. These results reveal that ·OH and ·O_2_^−^ are major species for degradation reactions in RhB or MB dyes^51^.

### Possible Mechanism

**Figure 6:**
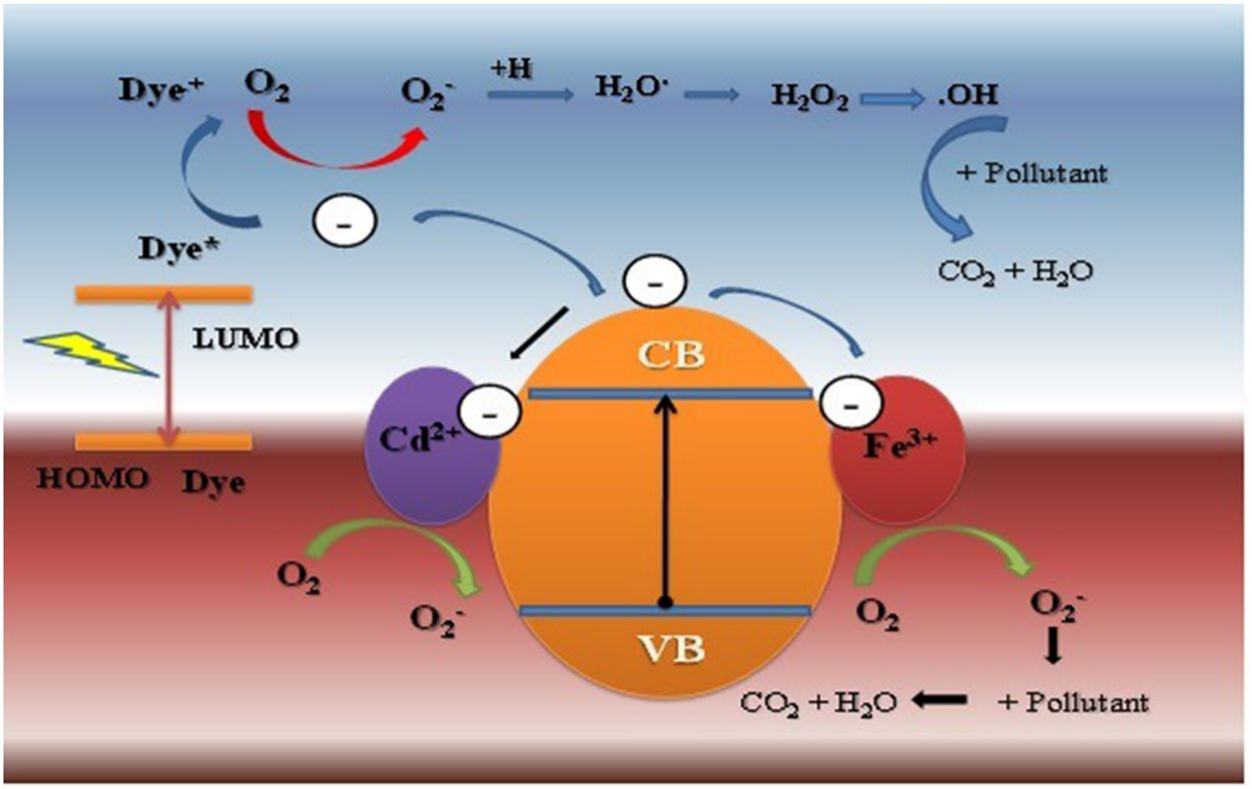
Schematic diagram for the charge separation and transfer of the photocatalysts under visible light irradiation.

It is evident that Fe-Cd co-doping almost has no significant influence on the morphology and crystal structure of the samples (as seen in TEM and XRD results). Although Fe-Cd co-doping influences the specific surface area (*S*BET) and crystal size of the samples to a certain extent, yet photocatalytic activity of the catalysts does not follow the trends in the variations of crystal size and *S*BET, completely. In order to explain the changes in photocatalytic activity under visible–light irradiation, the excitation and transfer process of charges between (Fe-Cd) and ZnO nanoparticles is schematically illustrated in Fig. 6. When Fe and Cd species were implanted in to the ZnO, an interaction between dye molecules and doped ZnO surface may takes place which serve a photodynamic mechanism of photo-oxidation. Therefore, it is believed that presence of Fe^3+^/Cd^2+^ species leads to increase the electron capture capacity for the pure ZnO photocatalysts from the photosensitizer.

Initially, under the influence of visible light (VL), the surface dye molecules may absorb the energy, and then introduced electrons into the conduction bands of ZnO nanoparticles. The surface photocurrent results show the ZnO cannot be excited by visible light. Therefore, self–sensitive decomposition of dye molecules is responsible for photocatalytic activity of pure ZnO. The excited dye molecules would inject electrons into the conduction bands of pure ZnO nanoparticles. For (Fe-Cd) co-doped ZnO, the conduction bands of ZnO or Fe/Cd ions received the electrons from excited dye [Eqs (1,2)]. The electrons injected into conduction band participate in in the photo–reaction processes described in equations chart. The Cd^2+^ can trap electron from dye molecules and take part in further process [Eqs (3,4)].

The Fe^3+^ ions trap electrons and convert into Fe^2+^. The Fe^2+^ ion is less stable so it reacts with oxygen molecules and changes again into Fe^3+^, reducing O_2_ to superoxide radical anion ·O_2_^−^ [Eqs (5,6)]. Furthermore, this superoxide radical anion ·O_2_^−^ reacts either with pollutant and degrades it [Eq. (7)] or reacts with H^+^ and takes part in further process [Eqs (8–11)].

Secondly, the electrons which comes from excited dye transferred to the conduction band of photocatalysts can react with electron acceptors such as O_2_ adsorbed on the surface of photocatalysts, thereby reducing it to the superoxide radical anion ·O_2_^−^ [Eq. (12)], which are further converted to ·OOH, H_2_O_2_ and ·OH species via a series of protonation, disproportionation and reduction steps [Eqs (8–11)]. The generation of ·OH radicals also plays a significant role in the photocatalytic reactions, as the ·OH radicals are the key reactive species in the Photocatalytic oxidation of RhB molecules.

Thirdly, as *t*_2g_ level of 3d orbital of Fe^3+^ ion located above the valence band of ZnO, it is quite feasible to absorb a photon (λ ≥ 400 nm) resulting in a Fe^4+^ ion and a ZnO conduction band electron. The Fe^4+^ reacts with surface hydroxyl group to produce hydroxyl radical, while conductive band electron interacts with adsorbed O_2_ to form ·O_2_^−^ species [Eqs (13,14), (7–12)]. Furthermore, a high concentration of Fe^3+^ions acts as the source of charge recombination for the photo-induced electrons and holes pair [Eqs (15–17)], which reduces the photocatalytic activity. In present case, the optimal doping concentration is 2% (1% Fe and 1% Cd) above which the Fe^3+^ ions steadily act as recombination centers and hence the photocatalytic activity gradually decreases. Thus the photo–generated superoxide ion (·O_2_^−^) and hydroxyl radical (·OH) are highly reactive in degrading the dye solution^52,53^. These processes not only accelerate the rate of interfacial charge transfer, but also enhance the generation of highly reactive oxidative species such as superoxide and hydroxyl radicals.

1 $${\rm{Dye}}+h{\nu }\to {{\rm{Dye}}}^{\ast }+{{\rm{e}}}^{-}\to {\rm{Dye}}\,+\,\cdot $$

2 $${{\rm{Dye}}}^{\ast }+{{\rm{e}}}^{-}+{\rm{Zn}}Np\to {{\rm{Dye}}}^{\ast }+{\rm{Zn}}Np({{\rm{e}}}^{-})$$

3 $${{\rm{Cd}}}^{2+}+{{\rm{e}}}^{-}\to {{\rm{Cd}}}^{+}({\rm{electron}}\,{\rm{trap}})$$

4 $${{\rm{Cd}}}^{+}+{{\rm{O}}}_{{\rm{2}}}\to {{\rm{Cd}}}^{2+}+{{{\rm{O}}}_{{\rm{2}}}}^{-}\,\cdot ({\rm{electronrelease}})$$

5 $${{\rm{Fe}}}^{3+}+{{\rm{e}}}^{-}\to {{\rm{Fe}}}^{2+}({\rm{electrontrap}})$$

6 $${{\rm{Fe}}}^{2+}+{{\rm{O}}}_{{\rm{2}}}({\rm{ads}})\to {{\rm{Fe}}}^{3+}+{{{\rm{O}}}_{{\rm{2}}}}^{-}\,\cdot ({\rm{electronrelease}})$$

7 $${{{\rm{O}}}_{{\rm{2}}}}^{-}\,\cdot +{\rm{Pollutant}}\to {\rm{Degradation}}\,{\rm{products}}$$

8 $${{{\rm{O}}}_{{\rm{2}}}}^{-}\,\cdot ++\,{\rm{H}}\to \cdot \,{\rm{OOH}}$$

9 $${\rm{2}}\,\cdot \,{\rm{OOH}}\to {{\rm{H}}}_{{\rm{2}}}{{\rm{O}}}_{{\rm{2}}}+{{\rm{O}}}_{{\rm{2}}}$$

10 $${{\rm{H}}}_{{\rm{2}}}{{\rm{O}}}_{{\rm{2}}}+{{\rm{e}}}^{-}\to \cdot \,{\rm{OH}}+{{\rm{OH}}}^{-}$$

11 $$\cdot {\rm{OH}}+{\rm{Pollutant}}\to {\rm{Degradation}}\,{\rm{products}}$$

12 $${{\rm{e}}}^{-}+{{\rm{O}}}_{{\rm{2}}}\to {{{\rm{O}}}_{{\rm{2}}}}^{-}\,\cdot $$

13 $${{\rm{Fe}}}^{3+}+h{\nu }\to {{\rm{Fe}}}^{4+}+{{\rm{e}}}^{-}$$

14 $${{\rm{Fe}}}^{4+}+{{\rm{OH}}}^{-}({\rm{ads}})\to {{\rm{Fe}}}^{3+}+\cdot \,{\rm{OH}}$$

15 $${{\rm{Fe}}}^{4+}+{{\rm{e}}}^{-}\to {{\rm{Fe}}}^{3+}$$

16 $${{\rm{Fe}}}^{2+}+{{h}}^{+}\to {{\rm{Fe}}}^{3+}$$

17 $${{\rm{Fe}}}^{2+}+\cdot {\rm{OH}}\to {{\rm{Fe}}}^{3+}+{{\rm{OH}}}^{-}$$

In summary, the photocatalytic activity of pure and Fe-Cd co-doped ZnO nanoparticles is studied by measuring the degradation of dyes RhB and MB as a test substance under the irradiation of visible light. The morphology studies and BET results show that the synthesized photocatalysts are spherical with large specific surface area (5.487–12.35 m^2^/g). The doping of Fe-Cd not only influences the surface area of nanoparticles, but also results in considerable enhancement of visible-light absorption and a red shift in the bandgap. The XPS results demonstrate that the Fe and Cd mainly exist in the form of Fe^3+^and Cd^2+^, and the binding energies of Zn-O bonds are altered with the Fe-Cd content. The photo-electrochemical studies indicate that the appropriate amount of loaded Fe-Cd (2 at.%), can effectively inhibit the recombination of photo-generated electron-hole pairs, improving the separation efficiency of charge carriers. The photocatalytic activity of the ZFC-x nanoparticles was investigated under visible light irradiation using MB and RhB dyes in aqueous solutions. It is found that ZFC-1 exhibits the highest photocatalytic activity owing to the large specific surface, which enhanced the light absorption and suppresses the charge recombination. These observations are in consistence with the photo-electrochemical investigations. An alternate mechanism for the enhancement of photocatalytic activity under visible light irradiation is proposed. The present results demonstrate successfully the photocatalytic performance of Fe-Cd co-doped ZnO nanostructures for the removal of pollutants, efficiently.

## Methods

### Materials

Zinc Nitrate hexahydrate Zn (NO_3)2_·6H_2_O ((>99%, Sigma), Iron (III) Nitrate nonahydrate Fe (NO_3_)_3_·9H_2_O ((>99%, Sigma), Cadmium (II) Nitrate hexahydrate Cd (NO_3_)_2_·6H_2_O ((>99%, Sigma), and citric acid [C_6_H_8_O_7_] (99.5% purity) were used directly without any further purification. Milli- Q water was taken to prepare all solutions.

### Preparation of pure and co-doped ZnO nanoparticles

Sol- gel route was used to synthesize Fe-Cd: ZnO with doping percentage of x (x = 0, 2, 4 and 6) (referred to as pure ZnO, ZFC-1, ZFC-2 and ZFC-3, respectively). In a typical synthesis process, first citric acid [C_6_H_8_O_7_] (99.5% purity) aqueous solution was prepared and then, appropriate proportion of analytical grade metal nitrates viz, Zn (NO_3_)_2_·6H_2_O (99.9% purity), Fe (NO_3_)_3_·9H_2_O (99.9% purity) and Cd (NO_3_)_2_·6H_2_O were thoroughly mixed in the citric acid solution while stirring it to get a homogeneous precursor solution. The precursor solution was kept at 80 °C for 3 h to obtain xerogel. The swelled xerogel was dried at 130 ^o^C for 12 h to get resultant powder. After grinding, the xerogel powders were sintered at 600 ^o^C for 10 h under air atmosphere to get pure and doped ZnO nanoparticles.

### Characterization of photocatalysts

The morphological features of the photocatalysts were characterized by scanning electron microscopy (SEM) (SIRION, FEI, Netherlands) with energy dispersive spectra (EDS), and transmission electron microscopy (TEM) (JEOLTEM-2010 (HT) instrument). The optical measurements were performed by UV–Vis absorption spectroscopy (PerkinElmer Lambda 725). The results of X-ray diffraction (XRD) were collected on Bruker D8 advance X-ray diffractometer by Cu K_α_ radiation and the accelerating voltage and current were 40 kV and 40 mA, respectively. The scans were acquired with a step of 0.02° in 2θ from 20° to 80°. The chemical composition of the samples and the valence states of various elements were analyzed by X-ray photoelectron spectroscopy (XPS, Thermo Fisher ESCALAB 250Xi). The C 1 s peak at 284.6 eV was used as a reference binding energy. The specific surface areas (SBET) were measured by nitrogen adsorption-desorption isotherm at 77 K using the instrument (JW-BK122W, China).

### Measurements of Photocatalytic Performance

The photocatalytic activities of all samples were performed by the degradation of an aqueous solution of RhB and MB (20 mg/L) at room temperature under visible light (VL) irradiation. In a typical experiment, the pure ZnO and ZFC-x (x = 1, 2 and 3) photocatalysts (100 mg) were dispersed in 100 mL RhB and MB aqueous solution (20 mg/L). To attain an adsorption–desorption equilibrium, the suspension was magnetically stirred under ambient conditions for 30 mins in dark environment. After reaching the adsorption-desorption equilibrium, the suspension was exposed to simulated solar light irradiation via a 300 W Xe lamp (172 mW/cm^2^, 15 cm away from the Photocatalytic reactor). For every 20 mins, 2 mL of the suspension was extracted and centrifuged (1200 rpm for 2 mins) to remove the photocatalyst. The degradation process was monitored by measuring the absorption of RhB and MB at ~553 and 664 nm respectively using a UV–Vis absorption spectrometer. A poly carbonate film was used as a UV cut off filter during VL irradiation.

## Electronic supplementary material

Supplementary Information
