# Supplementary material for: Enhanced visible light photodegradation activity of RhB/MB from aqueous solution using nanosized novel Fe-Cd co-modified ZnO
Source: Sci Rep. 2018 Jul 16;8:10691. doi: 10.1038/s41598-018-29025-1 (PMC6048152; doi:10.1038/s41598-018-29025-1)
Supplement: Supplementary file 1 — Supplementary Information [file 41598_2018_29025_MOESM1_ESM.docx]

**Enhanced visible light photodegradation activity of RhB/MB from aqueous solution using nanosized novel Fe-Cd co-modified ZnO**

Neena D.^1,2*#^, Kiran Kumar Kondamareddy^1,3*^, Han Bin^1^,Dingze Lu^4^,Pravin Kumar^5^, R.K. Dwivedi^6^, Vasiliy O. Pelenovich ^1^, Xing-Zhong Zhao^1^, Wei Gao ^2^, Dejun Fu^1#^

*^1^ Key Laboratory of Artificial Micro- and Nano-Materials of Ministry of Education, Wuhan University, and Hubei Key Laboratory of Nuclear Solid Physics, School of Physics and Technology, Wuhan University, Wuhan, China*

*^2^Department of Chemical & Materials Engineering, University of Auckland, Auckland, New Zealand*

*^3^Department of Physics, Veltech Rangarajan & Dr. Sagunthala R&D Institute of Science and Technology, Avadi, Chennai, Tamilnadu, India.*

*^4^Inter Department of Physics, Xi’an Polytechnic University, Xi’an, China*

*^5^Inter University Accelerator Centre (IUAC), Aruna Asaf Ali Marg, New Delhi, India*

*^6^Department of Physics, Christ Church College, Kanpur, India*

**Equal contribution*

*^#^corresponding author*

*E-mail address: djfu@whu.edu.cn (D.J. Fu), neena@whu.edu.cn (Neena D.)*


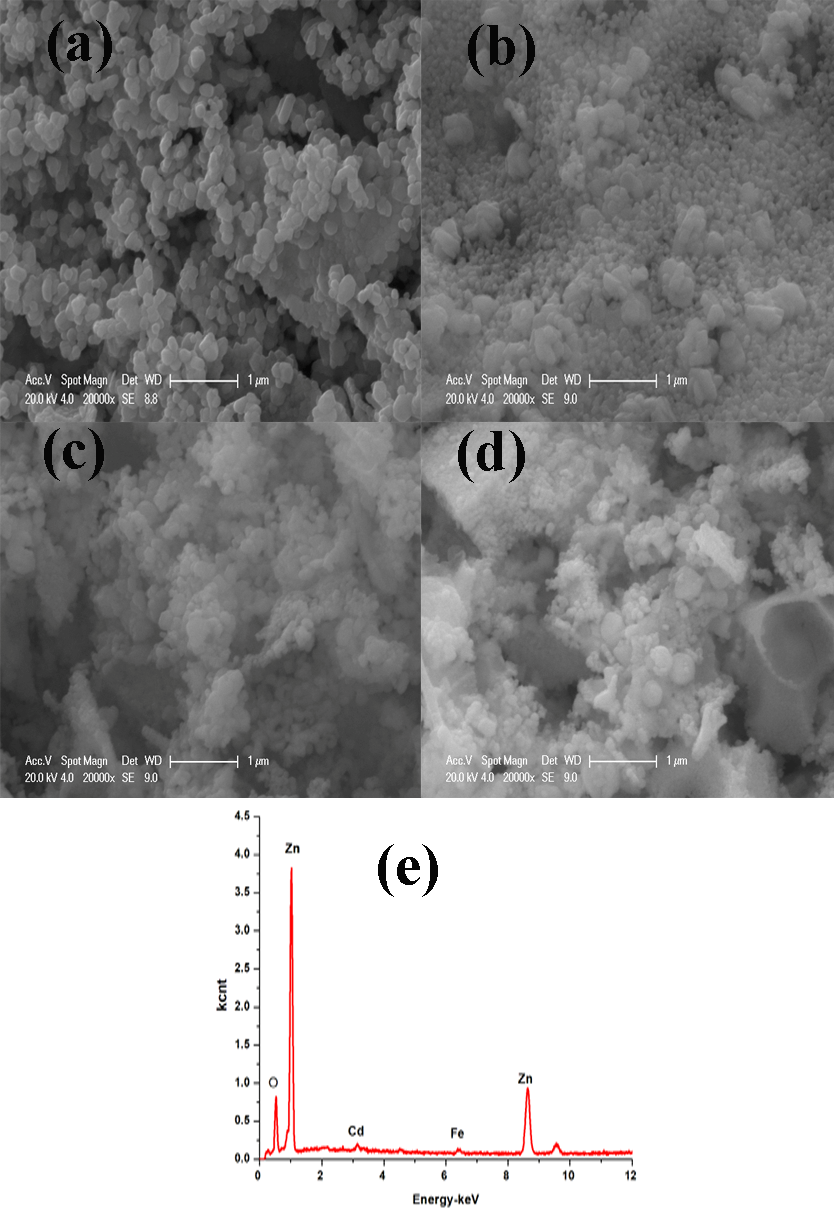


Figure S1: SEM micrographs for nanoparticles of (a) ZnO, (b) ZFC-1, (c) ZFC-2 and (d) ZFC-3 photocatalysts, (e) EDS for ZFC-x photocatalysts.


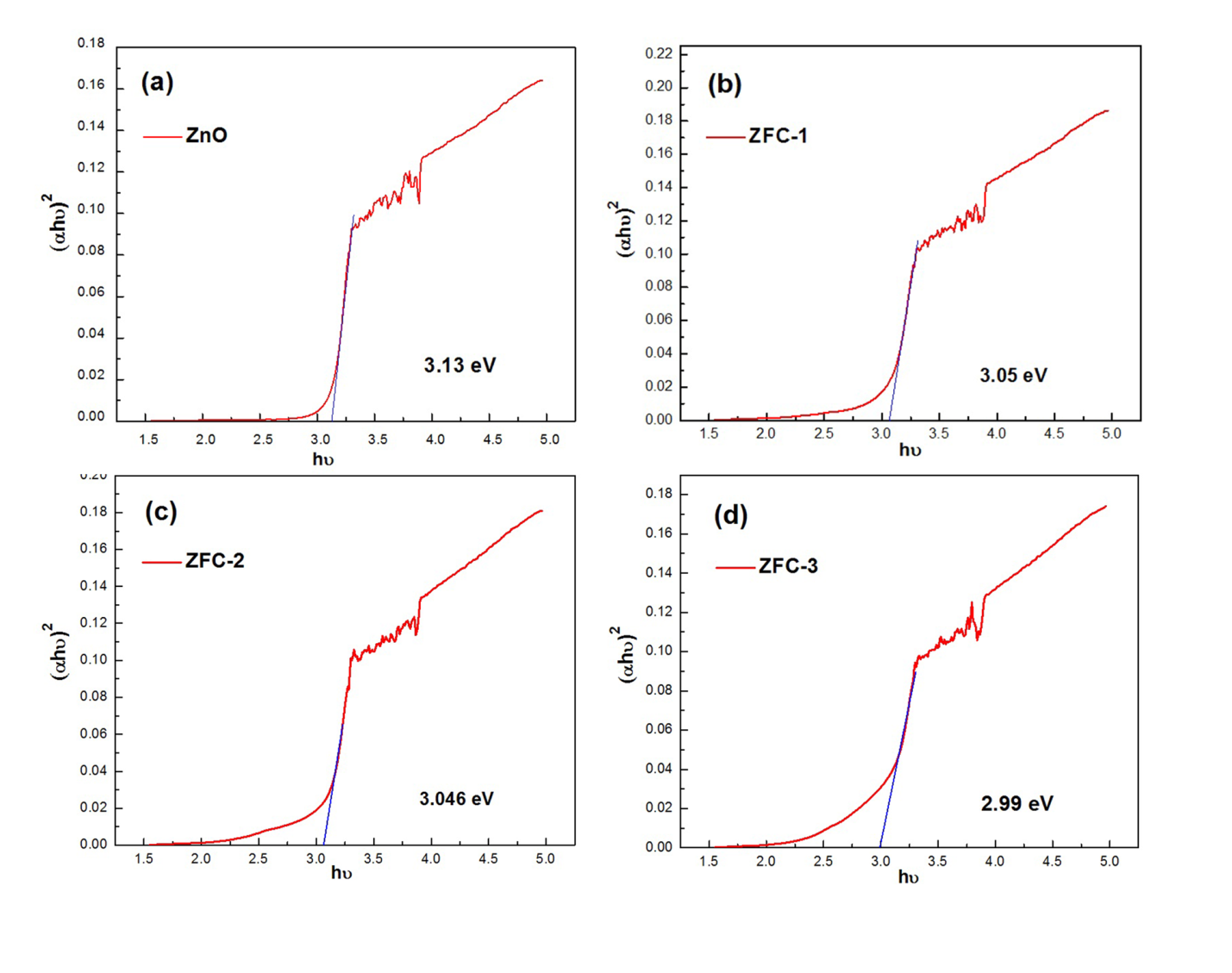


Figure S2: Bang gap for all samples


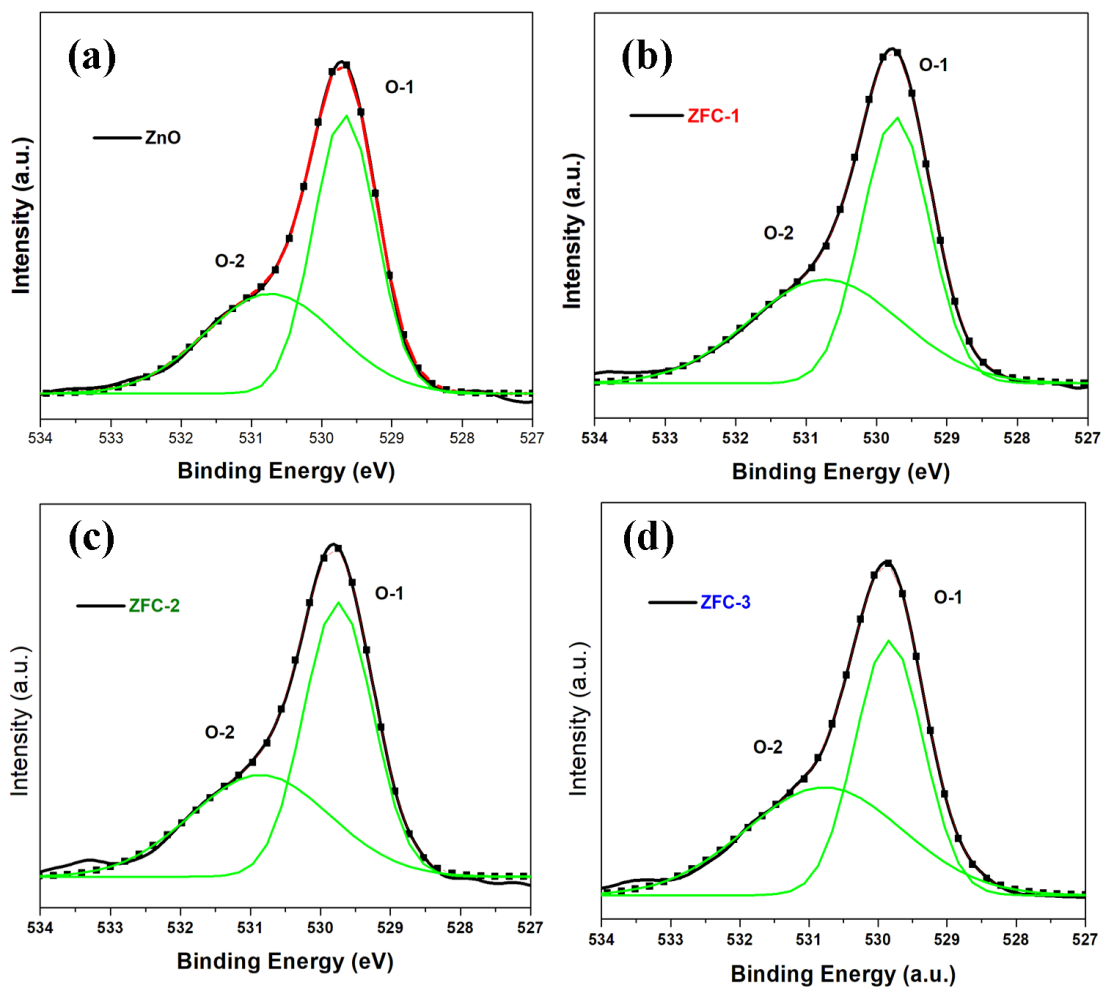


Figure S3: de-convoluted XPS scans of the O 1s core−electron region of all samples.


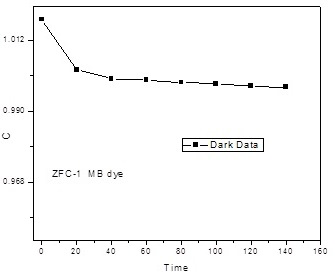


Fig S4: Adsorption experiment in dark for ZFC-1
